# Males have more aggressive and less sociable personalities than females in semi-captive Asian elephants

**DOI:** 10.1038/s41598-019-39915-7

**Published:** 2019-02-25

**Authors:** Martin W. Seltmann, Samuli Helle, Win Htut, Mirkka Lahdenperä

**Affiliations:** 1 Department of Biology, University of Turku, Turku, Finland; 2 Myanma Timber Enterprise, Department of Timber Extraction, MOECAF, Yangoon, Myanmar

## Abstract

Personality, i.e. consistent between-individual differences in behaviour, has been documented in many species. Yet little is known about how males and females of long-lived, highly social species differ in their measures of personality structure. We investigated sex differences in the mean, variance, and covariance of three previously reported personality traits (Attentiveness, Sociability, Aggressiveness) in 150 female and 107 male Asian elephants (*Elephas maximus*) from a semi-captive population in Myanmar. These three personality traits were obtained by performing exploratory factor analysis on 28 behavioural items that had been rated by experienced elephant handlers. We found that males scored significantly higher on Aggressiveness and tended to score lower on Sociability than females. However, no sex difference was found in the mean scores of Attentiveness. Variances for the three personality traits did not differ between the sexes, suggesting that male and female elephants share the same range of personality variation. Likewise, trait covariances were similar between the sexes. While both sexes show complex sociality in the wild, female Asian elephants typically live in highly social family units, whereas male elephants’ social bonds are weaker. Males usually form dominance ranks by aggressive interactions, especially during musth. Our results on a large sample of individuals living in their natural environment are thus in agreement with elephant life-histories and parallel the findings of sex differences in other long-lived highly social species with similar life-histories.

## Introduction

Many species show consistent between-individual differences in behaviour, termed (animal) personality^1,2^. Personality is biologically relevant, as differences in personality translate into differences in fitness^3,4^. Personality studies sometimes focus only on male or female individuals of a species, even though behaviour and life-history can differ substantially between the sexes, leading to different selection pressures on male and female personality^5,6^. However, researchers have recently been paying more attention to sex-specific differences in personality, advocating that sex differences should receive even more attention^7–9^, since ignoring them may potentially lead to flawed conclusions^8^. Males and females can differ consistently in their behaviour or personality since they face different selection pressures. For instance, males and females often follow different strategies for reproduction or mate choice. It has been shown that great tit (*Parus major*) males and females express different risk-taking behaviour in social and non-social contexts^10^. Furthermore, in zebra finches (*Taeniopygia guttata*), the sexes differ consistently in exploration, feeding, and leadership^6^.

Previously, we have identified three personality traits - Attentiveness, Sociability, and Aggressiveness - for 257 Asian elephants, derived by performing factor analysis on the ratings of 28 behavioural adjectives (e.g. active, playful, and moody) by experienced elephant handlers^11^. We also demonstrated that male and female elephants did not differ in personality structure. That is, the same number of personality traits were found for both sexes and these personality traits were related to the same behavioural adjectives in males and females. Despite uniformity in personality trait structure, group-differences in the trait means, variances, and covariances might still occur. For example, female white-faced capuchins (*Cebus capucinus*) score higher on Agreeableness^12^ and male chimpanzees (*Pan troglodytes*) score higher on Dominance^13^. In Asian elephants, females are in general more social and gregarious than males^14^, suggesting possible sex-specific differences in personality traits. In fact, a previous study investigating the personality structure of captive African (*Loxodonta africana*) and Asian elephants has demonstrated that at least female African elephants were rated as more agreeable (scoring higher on socio-positive behaviours) than males^15^. Therefore, our aim in this study is to examine sex differences in detail in pre-determined personality traits in a population of semi-captive Asian elephants living in their natural habitat.

This study system offers numerous advantages for addressing our aim. First, as adults, male and female elephants in the wild live almost completely separate lives, leading to sex-specific social structures and behaviours. Female Asian elephants live in small family units with strong bonds between the group members and group cohesion is of high importance^14^. Less is known about the social life of male Asian elephants: they can live solitary or in loose male groups and are more likely to be associated with females in oestrus during reproduction^14^. Males and females also differ, for example, in their foraging behaviour^16^, as well as in their physical appearance and development^14,17^, posing potentially different selection pressures on male and female personalities. Second, besides primates, little is known about personality differences between sexes in long-lived highly social mammals, as studies tend to focus on shorter lived species. Third, this study is based on a large sample of individuals who are not confined to artificial environments, and can thereby express a larger range of their natural behaviours. This study therefore provides an important addition to personality research, as it assesses sex differences in personality traits represented by 15 rated behaviours expressed among male and female elephants.

## Results

In order to compare means, variances, and covariances of the personality traits between the sexes, one should first confirm that the factors used to represent personality traits measure the same underlying concepts in both sexes. If not, the comparisons might be confounded by different measurement quality across the sexes^18^. This is called measurement invariance analysis, and it aims at establishing that the same constructs are truly compared among the sexes by setting sex-specific constraints to different model parameters depending on the level of invariance required for the specific comparison in question^19,20^. We thus started by conducting measurement invariance analysis between male and female elephants in a multi-group factor analysis framework^19^.

The measurement invariance analysis starts by confirming that the configural model with the same factor structure for both sexes (factor loadings, intercepts, and residual variances were freely estimated across the sexes) shows an adequate fit to the data. This was confirmed: both sexes in a multi-group factor analysis shared the same personality dimensions, indicated by an adequate model fit to the data (χ^2^ = 252.6, df = 174, p = 0.0001; RMSEA [90% Cis] = 0.051 [0.037, 0.065], p = 0.432; SRMR = 0.036; CFI = 0.975).

After confirming configural invariance between the sexes, metric and scalar invariances were evaluated to examine both the equality of factor loadings and of intercepts of corresponding items^19,20^. Metric invariance allows the comparison of factor variances and covariances between the sexes whereas scalar invariance allows the comparison of factor means between the sexes^19,20^. We found evidence for metric but not scalar invariance, although their effect sizes appeared rather small (<0.3) and accounted for a relatively low proportion of total variance (Table 1). Non-invariance of item intercepts was found only with respect to personality trait “Sociability”, where the items “friendly” and “playful” were found to be non-invariant between the sexes (Table 2). Since the personality trait “Sociability” has four other item intercepts that were found to be invariant, we should be able to reliably compare its mean between the sexes^21,22^.

**Table 1:** Tests and effect size measures for metric and scalar invariance.

Model comparison	Δχ^2^	df	p	*w*	% of variance explained
Metric against Configural	8.505	12	0.75	0.117	1.379
Scalar against Metric	29.216	12	0.0037	0.218	4.736

**Table 2:** The comparison of item intercepts using bias-corrected bootstrapping. Item intercepts whose 99% confidence intervals do not contain zero (i.e. non-invariant) are given in bold.

Personality trait	item	lower 0.5%	Difference est.	upper 0.5%
*Attentiveness*				
	attentive	−0.327	−0.087	0.175
	confident	−0.391	−0.124	0.133
	slow	−0.07	0.244	0.527
	active	−0.317	−0.05	0.204
	vigilant	−0.215	0.028	0.281
	obendient	−0.274	−0.011	0.207
*Sociability*				
	mischievous	−0.357	−0.096	0.135
	affectionate	−0.231	0.095	0.404
	**friendly**	**0**.**165**	**0**.**402**	**0**.**683**
	social	−0.132	0.114	0.351
	**playful**	**−0**.**547**	**−0**.**291**	**−0**.**022**
	popular	−0.503	−0.225	0.032
*Aggressiveness*				
	aggressive	−0.171	0.009	0.18
	dominant	−0.21	−0.046	0.129
	moody	−0.165	0.038	0.219

**Figure 1:**
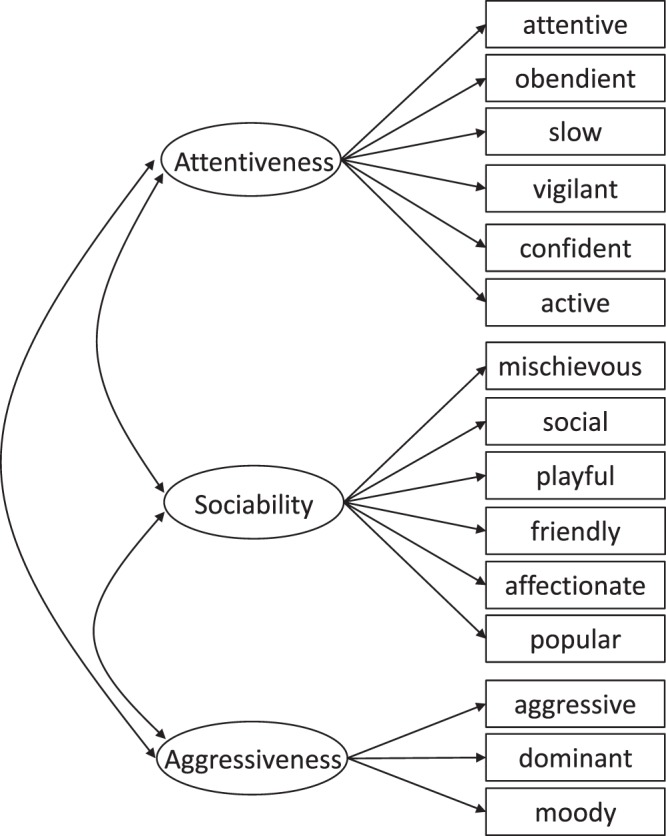
The 3 factor model for Asian elephant personality. Unidirectional arrows depict direct effects (regressions) of the latent variables (personality traits) onto the observed measures (rated behavioural items), whereas bidirectional arrows symbolize covariances between the personality traits.

We thus proceeded to compare trait means, variances, and covariances between the sexes. We found that males scored 0.27 (95% CIs = 0.03, 0.54) units higher on the “Aggressiveness” scale compared to females. Males also tended to be rated as less sociable than females, scoring 0.28 (95% CIs = −0.01, 0.61) units lower on “Sociability” scale than females (Fig. 1, Table 3). We found no sex difference in the means of the “Attentiveness” scale or in variances of any of the personality traits examined (Fig. 1, Table 3). In both sexes, “Attentiveness” positively covaried with both “Sociable” and “Aggressiveness” and “Sociable” positively covaried with “Aggressiveness” (Table 3). No sex differences were found with respect to these covariances among three personality traits (Table 3).

**Table 3:** Estimated means ($\bar{{\rm{X}}}$), variances (σ) and covariances (ϕ) of the three personality traits in female and male Asian elephants, and their sex differences.

	Females	Males	Difference
$\bar{{\rm{X}}}$_Attentiveness_	4.265 (4.044, 4.510)	4.431 (4.134, 4.709)	−0.166 (−0.500, 0.223)
$\bar{{\rm{X}}}$_Sociability_	3.458 (3.280, 3.678)	3.176 (2.933, 3.438)	0.282 (−0.009, 0.611)
$\bar{{\rm{X}}}$_Aggressiveness_	2.062 (1.921, 2.237)	2.334 (2.143, 2.556)	−0.272 (−0.542, −0.034)
σ_Attentiveness_	2.713 (2.391, 3.060)	3.084 (2.583, 3.578)	−0.371 (−0.945, 0287)
σ_Sociability_	1.833 (1.548, 2.155)	1.846 (1.459, 2.229)	−0.013 (−0.496, 0.474)
σ_Aggressiveness_	1.078 (0.806, 1.457)	1.092 (0.796, 1.515)	−0.013 (−0.555, 0.432)
ϕ_Attentiveness, Sociability_	1.960 (1.714, 2.222)	1.758 (1.356, 2.179)	0.202 (−0.296, 0.653)
ϕ_Attentiveness, Aggressiveness_	1.077 (0.903, 1.283)	1.075 (0.850, 1.303)	0.002 (−0.283, 0.267)
ϕ_Sociability, Aggressiveness_	0.829 (0.658, 1.032)	0.765 (0.613, 0.971)	0.064 (−0.185, 0.327)

The numbers in parentheses are 95% bias-corrected bootstrap confidence intervals from 1,000 samples.

## Discussion

Our study, using 15 rated behaviours from a large sample of Asian elephants living in their natural habitat, provides an important addition to the growing literature on sex differences in personality, especially in long-lived highly social mammals. In species where life-histories differ between the sexes, we expect selection to act differently on sexes, leading to differences in behaviour and personality^5^. While the personality trait structure and the covariances between the personality factors seem to be the same for male and female Asian elephants in our study population^11^, we still find differences in the mean scores in Sociability and Aggressiveness between male and female individuals. However, the variances of the three personality traits did not differ between the sexes, suggesting that male and female elephants share the same range of personality variation. For both males and females, covariances between personality traits were positive, indicating that individuals who were scored higher on Attentiveness were also scored higher on Sociability and Aggressiveness, and individuals who were scored higher on Sociability were also scored somewhat higher on Aggressiveness.

Female elephants had a tendency to score higher on Sociability than males, indicating that they may be more affectionate and both actively seek out others and are sought out by others as company. Females were also rated as showing more socio-positive behaviour towards people and elephants outside of their working group. Higher female agreeableness appears to be a common pattern in long-lived highly social mammals (humans^23^; chimpanzees^13^), and has been observed before in Asian and African elephants in zoos^15^. These sex-specific differences in elephant Sociability could be explained by the different social lives of the sexes. In general, in species where males are dispersing from the natal group and family units are made up by related females, it is more likely that females form stronger long-term social bonds and closer associations^24^. Female Asian elephants live in small, strongly bonded family units and group cohesion is of high importance^14^. Members of a family unit exhibit a linear age-structured dominance hierarchy and the social rank is an important mediator in avoiding within-group conflict over resources (e.g. Moss *et al*.^25^). Exhibiting consistent and predictable behavioural types or personalities could further improve the resolution of within-group conflict, with different personalities adopting different social roles when confronted with social challenges^26^. Female white-faced capuchin monkeys were also found to be more agreeable than males^12^, suggesting a possible influence on mothering and allomothering behaviours. This might be true for female elephants as well, as the presence of mothers and grandmothers have positive effects on calf survival and reproduction^27,28^. Little is known about the social life of male Asian elephants, however, which tended to score lower on Sociability compared to females. Male African elephants need to develop social skills as well since they live in a complex bull society^29^ and they seek out companions when not sexually active^30^. However, these bonds are not as strong as in females^31^, and such males have been suggested to associate with other males to determine each other’s status or dominance prior to going into musth^30^. Male Asian elephants, on the other hand, often live solitarily or in loose male groups, and are more likely associated with females in oestrus during musth^14^. Close relationships with other individuals may therefore serve different purposes for males and females^29,32^.

We also discovered that males scored higher on the Aggressiveness scale than females, with similar patterns found in chimpanzees^13^, in which males score higher on Dominance and are rated as more aggressive, and to some extent in humans^23^ (though here men scored higher on assertiveness rather than aggression itself). Higher Aggressiveness in male elephants might be explained by their need to assess each other’s dominance status by less-aggressive sparring bouts or by more-aggressive interactions during musth^14^. The Aggressiveness personality trait is related to ratings of aggressive, moody, and dominant behaviour^11^ (Fig. 1), and higher aggression has a crucial impact in maximising reproductive success in male elephants as older larger and more aggressive males are more successful in mate guarding than less aggressive males^33^.

The personality trait Attentiveness includes behaviours that are generally associated with the response of the elephant towards its mahout and how the elephant acts in its working environment, like attentive, obedient, and vigilant^11^ (Fig. 1). Since both sexes work and live under similar conditions and similar work regulations apply to them (e.g., number of work days, number of logs), it is not surprising that those behaviours are not significantly influenced by sex.

Although mortality rates^34^, reproductive profiles^35^, and social behaviours^28^ of Myanmar timber elephants resemble those of truly wild elephants, we would like to point out that our results on personality sex differences may not be directly applicable to truly wild elephants. Aside from one study on female wild African elephant personality^36^, almost nothing is known about wild elephant personality. The behavioural repertoire of timber elephants is probably influenced by their interactions with humans and the semi-captive living conditions, and the Attentiveness factor, observed for the first time in our study population^11^, could be either unique for our study population or to working elephants in general. Another limitation of our study is the potential bias of a rater’s personality, personal attitude and experiences with elephants on the ratings of elephants’ behaviours. Previous studies have demonstrated that pet owners’ personality is related to pets’ behaviour^37^. Hence, it is possible that mahouts’ own personalities might relate to how they rate their elephant’s personality, and we suggest that future research should investigate this link to obtain clarity about this potential bias. In addition, age might be a confounding factor when looking at sex differences in personality, since personality and social bonds can change between life stages^13,29,32,38^. However, we are unable to address this issue with our current dataset, which comprises four years of data of a long-lived mammal. Even though age-dependent changes could be present, they would not affect individual personality in such a short timeframe, relative to the longevity of elephants. Furthermore, the same measurement invariance assumption that we are examining here with respect to sex also concerns individual age. Testing for measurement invariance for individuals of different ages in both sexes would require the categorisation of age, which would lead to an unacceptably low sample size for juvenile elephants. Therefore, more data are needed to investigate longitudinal changes in elephant personality, and we are in the process of collecting more data and will hopefully be able to address this problem in the future.

Our study contributes to the growing literature on the existence of sex differences in animal personality. We found evidence for sex differences in two of the three personality traits in this population of Asian elephants, and these differences are in line with previous findings on other long-lived highly social mammals and on elephants investigated in captive and wild environments. Such differences are still not commonly reported, and we encourage researchers to include both sexes in personality studies whenever possible. Males and females of the same species can experience different selection pressures and follow different life-history strategies, which in turn can be reflected by sex-specific personality differences.

## Methods

### Subjects

Our study included 150 female and 107 male semi-captive Asian elephants living in their natural habitat and ranging in age from 3 to 76 years (females: 3–76, median: 29.5; males: 5–65, median: 16). These elephants are timber elephants from Myanmar, and their life-histories closely resemble those of wild elephants^28,34^. Elephants work during government-set working hours where they are used during the day as riding, transport, and draft animals. At night the elephants forage unsupervised in nearby forests where they encounter tame and wild conspecifics, hence the ‘semi-captive’ definition of these animals. The three personality traits, Attentiveness, Sociability, and Aggressiveness, have previously been evaluated in our study population by means of factor analysis (Fig. 1)^11^. This study was based on questionnaires collected from 2014 to 2017 for which two experienced elephant riders (mahouts) rated the frequency of 28 behavioural items of elephants on a 4-point scale, with 1 meaning ‘Very rarely’, 2 ‘Occasionally’, 3 ‘Quite a lot’ and 4 ‘Most of the time’^11^. Figure 1 shows the final factor model, with 15 behavioural items loading on the three personality traits, Attentiveness, Sociability, and Aggressiveness. We used summed ratings per measurement occasion for each elephant, resulting in a range of 1 to 8, which are the corresponding units referred to in the results. National governmental authorities and the ethical board of the University of Turku approved the research. All methods were performed in accordance with relevant guidelines and regulations.

### Statistical analysis

Our analysis begun by conducting measurement invariance analysis between male and female elephants in a multi-group factor analysis framework^19^. All the items measuring the particular personality trait were treated as continuous variables; for more details, please see^11^. Since the item scores were recorded on the same scale, we used effects-coding method to identify our factors: that is, factor loadings and intercepts were constrained to have average values of 1 and 0, respectively^39^. This means that factor means and variances are interpreted as optimally weighted averages of their items and as the average amount of items’ variance explained by the given personality trait, respectively^39^. We started by first confirming that the configural model (i.e. an unconstrained model where factor loadings, intercepts, and residual variances are free across groups and only the factor structure is the same among groups) shows an adequate fit to the data. Model fit to the data was examined using the exact chi-square test (χ^2^) and the following fit-indices: the root mean square error of approximation (RMSEA), standardized root mean square residual (SRMR), and the comparative fit index (CFI). Both RMSEA and SRMR are badness-of-fit measures, where 0 indicates a perfect fit for the model. In contrast, a value approaching 1 in CFI indicates good model fit. RMSEA has the added benefit of providing 90% confidence intervals for the estimate and it can be used to test the null hypothesis that the estimate is <0.05, indicating a good fit. The rough cut-off values used to indicate a well-fitting model for SRMR and CFI are <0.08 and >0.95, respectively.

Second, if configural invariance holds among the groups examined, metric and scalar invariances were evaluated to examine the equality of factor loadings and intercepts of corresponding items between the sexes, respectively^19,20^. Metric invariance allows the comparison of factor variances and covariances between the sexes, while scalar invariance allows the comparison of factor means between the sexes^19,20^. We used chi-square difference (or likelihood ratio) test for nested model comparisons and effect size measures, as given in Newsom^40^. These were *w* effect size measures, which can be interpreted along the lines of general effects size measures suggested by Cohen^41^. Moreover, we used the percent of variance explained by the level of invariance^40^. The comparisons were made between configural vs. metric invariance models and between metric vs. scalar invariance models. If metric or scalar invariance is found, the identification of non-invariant items was done by comparing each individual item intercept and loading between males and females using bias-corrected bootstrapping using 1,000 draws. In order to control for Type I error rate, 99% bootstrap confidence intervals were used^42^. Item intercepts or loadings were considered to be invariant between the sexes if these confidence intervals include zero^42^. If such non-invariance concerns only a minority of items, one can assume partial invariance and proceed to mean, variance, and covariance comparisons by freely estimating those few non-variant items by sex^21,22^. The potential differences in factor means, variances, and covariances between the sexes were estimated using 95% bias-corrected bootstrap confidence intervals from 1,000 samples. Because our data comprises repeated ratings of some individuals, we used a design-based clustering method that corrects for non-independence of data points by adjusting parameter SEs without explicitly estimating this dependency in all analyses^43^. Missing data were handled with full information maximum likelihood (FIML). All analyses were conducted using Mplus version 8.2 using robust maximum likelihood estimator^44^.

## Supplementary information

Dataset 1

## Data Availability

The data is provided as electronic Supplementary Material “ESM_SeltmannEtAl_data”.
